# Spatial and functional dissection of cancer-associated fibroblasts-mediated immune modulation in *H. pylori*-associated gastric cancer

**DOI:** 10.1186/s12943-025-02490-9

**Published:** 2025-11-06

**Authors:** Bonan Chen, Hongzhen Tang, Xiaohong Zheng, Fuda Xie, Peiyao Yu, Yang Lyu, Tiejun Feng, Jialin Wu, Jingya Liu, Yi Xu, Alvin H. K. Cheung, Canbin Fang, Zhangding Wang, Shouyu Wang, Justin Chak Ting Cheung, Yujuan Dong, Ruoxi Tian, Yigan Zhang, Cheng Lu, Chi Chun Wong, Jun Yu, William K. K. Wu, Elke Burgermeister, Man Tong, Fengbin Zhang, Wei Kang, Kam Tong Leung, Ka Fai To

**Affiliations:** 1 Department of Anatomical and Cellular Pathology, State Key Laboratory of Translational Oncology, Sir Y.K. Pao Cancer Center, The Chinese University of Hong Kong, Hong Kong SAR, China; 2 State Key Laboratory of Digestive Disease, Institute of Digestive Disease, Li Ka Shing Institute of Health Science, The Chinese University of Hong Kong, Hong Kong SAR, China; 3 CUHK-Shenzhen Research Institute, Shenzhen, China; 4 School of Life Science and Technology, Harbin Institute of Technology, Harbin, China; 5 The Fourth Clinical Medical College of Guangzhou University of Chinese Medicine, Shenzhen, China; 6 Guangzhou University of Chinese Medicine, Guangzhou, China; 7 Department of General Surgery, The Second Affiliated Hospital of Harbin Medical University, Harbin, China; 8 Department of Gastroenterology, The Affiliated Drum Tower Hospital of Nanjing University Medical School, Nanjing, China; 9 Department of Surgery, The Chinese University of Hong Kong, Hong Kong SAR, China; 10 Institute of Biomedical Research, Taihe Hospital, Hubei University of Medicine, Shiyan, China; 11 Department of Radiology, Guangdong Provincial People’s Hospital, Medical Research Institute, Southern Medical University, Guangzhou, China; 12 Department of Medicine and Therapeutics, The Chinese University of Hong Kong, Hong Kong SAR, China; 13 Department of Anaesthesia and Intensive Care, The Chinese University of Hong Kong, Hong Kong SAR, China; 14 Department of Medicine II, Medical Faculty Mannheim, University Medical Center Mannheim, Heidelberg University, Mannheim, Germany; 15 School of Biomedical Sciences, The Chinese University of Hong Kong, Hong Kong SAR, China; 16 Department of Gastroenterology, The Fourth Hospital of Hebei Medical University, Shijiazhuang, China; 17 Department of Pediatrics, The Chinese University of Hong Kong, Hong Kong SAR, China

**Keywords:** Cancer-associated fibroblasts, Gastric cancer, Spatial transcriptomics, *H. pylori*, Lauren classification

## Abstract

**Background:**

Cancer-associated fibroblasts (CAFs) are key regulators of the tumor microenvironment, yet their spatial organization and immunomodulatory functions in *H. pylori*-associated gastric cancer (GC) remain incompletely understood.

**Methods:**

We profiled formalin-fixed paraffin-embedded (FFPE) tumors from 71 GC patients using spatial transcriptomics and integrated single-cell RNA-seq from three independent cohorts (China, USA, and Singapore). CAF-immune cell colocalization was quantified by neighborhood enrichment and aggregation index score. Ligand-receptor inference and trajectory analysis resolved CAF signaling and state transitions. To delineate post-transcriptional control, ARE-motif scanning and expression correlations were combined with laser-assisted crosslinking and immunoprecipitation sequencing (LACE-seq) to nominate ZFP36 targets. Immune contexture and prognostic associations were evaluated using CIBERSORT-ABS and Kaplan-Meier analyses in The Cancer Genome Atlas (TCGA) and Asian Cancer Research Group (ACRG) cohorts.

**Results:**

We first defined the spatial distributions of the four CAF subtypes reported in prior studies and found that their immune associations varied across histologic and infection-defined GC subtypes. In *H. pylori*-positive tumors, THBS1⁺ CAFs were spatially enriched near regulatory T cells (Tregs) and were associated with local immunosuppression through WNT5-FZD interactions. In parallel, ZFP36 bound AU-rich elements within the FN1 3′ untranslated region (3'UTR), destabilizing FN1 mRNA and thereby diminishing FN1⁺ CAF-mediated cytotoxic T lymphocyte (CTL) activation. Together, these axes promoted Treg accumulation and suppression of CTL activation.

**Conclusions:**

These findings reveal infection-associated stromal programs that shape the immune landscape in GC and highlight CAF-directed pathways as potential therapeutic targets in *H. pylori*-associated GC.

**Graphical abstract:**

***H. pylori*-induced CAF-mediated immunosuppression in GC. **1. *H. pylori* infection triggers transcriptional reprogramming of CAFs within the tumor microenvironment of GC. 2. The CAF subset marked by THBS1 is spatially enriched adjacent to Tregs and facilitates their recruitment via WNT5-FZD ligand-receptor signaling. 3. The RNA-binding protein ZFP36 binds AU-rich elements in the FN1 3’UTR, destabilizing FN1 mRNA and thereby attenuating CTL activation. 4. The THBS1-WNT5 axis stabilizes Tregs, whereas the ZFP36-FN1 axis limits CTL activation, converging to drive local immunosuppression in *H. pylori*-positive GC.

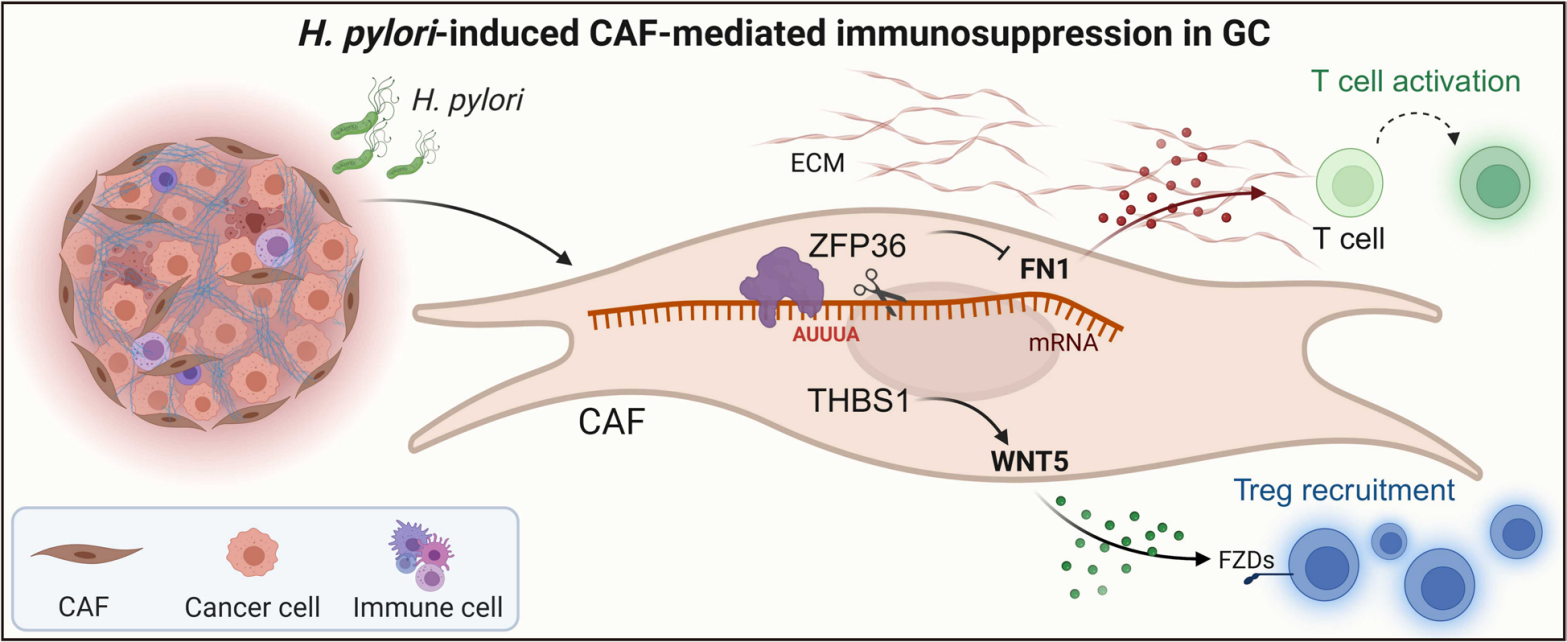

**Supplementary Information:**

The online version contains supplementary material available at 10.1186/s12943-025-02490-9.

## Background

Gastric cancer (GC) is a major global health burden and remains one of the leading causes of cancer-related mortality worldwide, with limited improvements in prognosis despite therapeutic advances [1]. Its development reflects a complex interplay among genetic, environmental, and microbial factors, with *Helicobacter pylori* (*H. pylori*) recognized as a well-established driver of gastric tumorigenesis [2, 3]. Particularly, *H. pylori* impose a disproportionate burden in Asia, exhibiting high regional prevalence and the strongest epidemiological linkage to gastric carcinogenesis [4]. By inducing chronic inflammation, epithelial injury, and dysplasia, *H. pylori* perturbs stromal and immune compartments, thereby disrupting gastric tissue homeostasis and laying the foundation for malignant transformation [5, 6].

Histologically, GC has long been classified by the Lauren system into intestinal-type and diffuse-type subtypes, which differ not only in morphology and clinical course but also in their response to *H. pylori*-induced inflammation [7–9]. Intestinal-type GC is more tightly associated with chronic atrophic gastritis and intestinal metaplasia, with higher *H. pylori* detection rates than diffuse-type GC [10]. Conversely, diffuse-type GC appears more influenced by genetic predisposition and less dependent on infection-mediated inflammation. These contrasts suggest subtype-specific routes to tumor development [11, 12], yet the precise impact of *H. pylori* on immune composition and the molecular features of each subtype remains insufficiently defined.

A key consequence of *H. pylori* infection is the persistent activation of the gastric immune microenvironment. This is characterized by immune cell infiltration and sustained secretion of pro-inflammatory cytokines, which not only damage the epithelium but also modulate stromal cell behavior, particularly that of cancer-associated fibroblasts (CAFs) [2]. CAFs have emerged as key mediators of *H. pylori*-driven inflammation. Preclinical studies have shown that *H. pylori* can induce the differentiation of normal fibroblasts into CAF-like cells [13], and our previous work further demonstrated that *H. pylori* promotes CAF recruitment and extracellular matrix (ECM) remodeling via the NF-κB/PIEZO1/YAP1/CTGF signaling axis [14]. Once activated, these CAFs secrete a broad array of cytokines, chemokines, and ECM components that collectively promote epithelial proliferation, angiogenesis, and immune evasion [15–17]. Recently, we systematically characterized the CAF compartment and identified four transcriptionally and functionally distinct subtypes, including progenitor CAF (proCAF), inflammatory CAF (iCAF), myofibroblastic CAF (myCAF), and matrix CAF (matCAF) [18]. While this classification highlights the functional heterogeneity of CAFs, it remains unclear how *H. pylori* infection shapes the composition of these subtypes, and whether their spatial interactions with cancer cells differ across GC histological subtypes.

Obviously, spatial transcriptomics, as an emerging technology, has rapidly become a powerful tool for decoding the complex spatial architecture of the GC tumor microenvironment (TME), enabling the localization of gene expression while preserving the integrity of tissue structure [19]. Furthermore, when integrated with single-cell RNA sequencing (scRNA-seq), it allows for high-resolution characterization of cellular heterogeneity, developmental trajectories, and intercellular communication [20]. In this study, we employed a combined spatial and single-cell transcriptomic strategy to systematically characterize the spatial organization of CAFs across different subtypes of GC, and to evaluate the impact of *H. pylori* infection on CAF composition and immunoregulatory functions, with the goal of elucidating the role of CAFs in shaping the immunosuppressive microenvironment in GC.

## Methods

### GC patient cohorts and cell lines

This study incorporated multiple GC patient cohorts across diverse geographic and clinical settings to support comprehensive spatial and single-cell transcriptomic analyses. For spatial transcriptomic profiling, formalin-fixed paraffin-embedded (FFPE) tumor samples were collected from 71 GC patients diagnosed between 1999 and 2006 at the Prince of Wales Hospital, Hong Kong. Ethical approval for this cohort was obtained from the Joint Chinese University of Hong Kong-New Territories East Cluster Clinical Research Ethics Committee (CREC Ref. No. 2022.060). scRNA-seq data were obtained from three independent GC cohorts. The China cohort included 10 GC patients recruited from the Affiliated Drum Tower Hospital of Nanjing University Medical School, with ethics approval granted by the institutional review board [21]. The USA cohort comprised 22 GC cases from a previously published study (https://dna-discovery.stanford.edu/research/datasets/) [22]. The Singapore cohort included 26 cases and was accessed through the Gene Expression Omnibus database (GSE183904) [23]. Besides, the human GC cell line HGC-27 was obtained from ECACC (Cat. 94,042,256) and authenticated by short tandem repeat (STR) profiling prior to use. Cells tested negative for mycoplasma by routine screening. HGC-27 cells were maintained in RPMI-1640 supplemented with 10% fetal bovine serum and 1% penicillin-streptomycin at 37°C in 5% CO₂. HGC-27 was used exclusively for LACE-seq experiments, and cells were cultured under the same conditions immediately prior to UV crosslinking and library preparation.

### Spatial transcriptomic profiling using cosMx SMI

FFPE GC tissue sections from 71 patients were processed for spatial transcriptomic analysis. Sections were mounted on CITOGLAS slides and incubated overnight at 60°C to ensure optimal tissue adherence. Heat-induced epitope retrieval (HIER) was performed using ER1 buffer (Leica Biosystems) at 100°C for 15 min, followed by enzymatic digestion with proteinase K (in ACD Protease Plus) for 30 min. After rinsing with DEPC-treated water, fiducial markers (Bangs Laboratories) diluted in 2 × SSCT were applied for spatial alignment, followed by washing in PBS and fixation in 10% neutral-buffered formalin. Slides were then washed with Tris-glycine and PBS, blocked with 100 mM NHS-acetate at room temperature, and rinsed in 2 × SSC. A SecureSeal hybridization chamber (Grace Bio-Labs) was placed onto each slide.

After brief denaturation at 95°C, hybridization was performed overnight at 37°C using a probe mixture containing 980-plex and custom-designed ISH probes (NanoString), attenuation probes, buffer R, and SUPERase·In. Post-hybridization, slides were sequentially washed with 2 × SSCT, 50% formamide in 2 × SSC at 37°C, and 2 × SSC at room temperature. Slides were re-blocked and prepared for imaging on the CosMx™ Spatial Molecular Imager (NanoString Technologies). Fields of view (FOVs; 0.51 × 0.51 mm) were selected based on H&E-stained reference sections. Reporter probes were introduced into the flow cell and imaged after incubation and washing. Nine-layer Z-stack images were acquired at 0.8 μm intervals for each FOV. For spatial protein visualization, tissue sections were stained with fluorophore-conjugated antibodies against PanCK, CK8/18, CD45, membrane, and nucleus, followed by multichannel fluorescence imaging. Image data were processed using the AtoMx Spatial Informatics Platform for downstream analysis.

### Image processing and spatial transcript assignment

High-content image analysis was performed to segment cells and assign RNA transcripts to spatial coordinates [24]. Cellular boundaries were identified using Z-stack immunofluorescence images, including nuclear (DAPI) and membrane signals, through machine learning-based segmentation algorithms. Detected transcripts were then spatially mapped to individual cells and their subcellular compartments, enabling the reconstruction of transcriptomic profiles at single-cell resolution. To facilitate visualization, transcript positions were overlaid with segmented cell contours using the napari image viewer. Distinct pseudocolors were applied to each immunofluorescence channel, and image contrast was optimized to generate composite visualizations of RNA expression and tissue architecture.

### ScRNA-seq data processing

Single-cell transcriptomic data were processed using Python (v3.8), 3 public single-cell datasets were integrated by invoke “anndata.concat”. Cells with fewer than 500 counts, fewer than 200 detected genes, or mitochondrial gene expression exceeding 20% were excluded to remove low-quality or stressed cells. Downstream analyses were performed using the Scanpy(V1.9.6). Raw counts were normalized and scaled with the “scanpy.preprocessing.normalize_total” and “scanpy.preprocessing.scale” functions. To correct for batch effects across samples, the Harmony integration method was applied.

### Cell type annotation

Dimensionality reduction was conducted using principal component analysis (PCA), followed by graph-based clustering with “scanpy.tools.leiden” and dimensionality reduction with “scanpy.tools.umap”. Uniform manifold approximation and projection (UMAP) was used for visualization. Differentially expressed genes (DEGs) were analyzed with “scanpy.tools.rank_gene_groups”, and cell type identities were assigned based on canonical marker genes and published cell type-specific transcriptional signatures.

### Neighborhood enrichment analysis

To evaluate spatial colocalization patterns between different cell types, neighborhood enrichment analysis was performed using the spatial coordinates and cell-type annotations derived from CosMx SMI data. For each cell, the frequency of neighboring cell types within a defined spatial radius was quantified. A permutation-based statistical framework was used to compare the observed co-occurrence frequencies against a null distribution generated by random shuffling of cell-type labels across spatial coordinates. Enrichment scores were calculated as log-transformed observed-over-expected ratios. The resulting enrichment matrix was visualized as a dot plot, with color intensity representing enrichment strength and dot size indicating significance level. This analysis enabled the identification of preferential spatial interactions between CAF subtypes and surrounding cell populations.

### Spatial Aggregation Index (SAI) score calculation

To quantify the spatial relationship between different cell types, a distance-penalized SAI score based on neighborhood composition and inter-cell distances was developed. Spatial coordinates of all cells were extracted from the AnnData object (adata.obsm[‘spatial’]). For each cell *i*, we identified its *t* nearest neighbors (Euclidean metric; NearestNeighbors). Within the neighborhood of *i*, cells annotated as the center or target type were flagged, and the following quantities were defined: (a) $N_{\mathrm{center}}^{\mathrm{global}}$: total number of center cells in the tissue. (b) $M_{\mathrm{target}}^{\mathrm{global}}$: total number of target cells in the tissue. (c) $n_{i,\mathrm{center}}^{\mathrm{local}}$: number of center cells among the neighbors of a given cell. (d) $m_{i,\mathrm{target}}^{\mathrm{local}}$: number of target cells among the neighbors. (e) ${d}_{i}$: mean pairwise Euclidean distance between local center and target cells in the neighborhood of *i*. For each neighborhood, the mean pairwise Euclidean distance between local center and target cells was computed. To penalize large separations, the reciprocal of this mean distance was taken as the distance weight. The SAI score was then defined as:

$${\mathrm{SAI}}_i=\log\left(1+\frac Tt\cdot\frac{m_{i,\mathrm{target}}^{\mathrm{local}}}{n_{i,\mathrm{center}}^{\mathrm{local}}}\cdot\sqrt{\frac{m_{i,\mathrm{target}}^{\mathrm{local}}\cdot n_{i,\mathrm{center}}^{\mathrm{local}}}{M_{\mathrm{target}}^{\mathrm{global}}\cdot N_{\mathrm{center}}^{\mathrm{global}}}}\cdot\frac{n_{i,\mathrm{center}}^{\mathrm{local}}+m_{i,\mathrm{target}}^{\mathrm{local}}}t\cdot\frac1{d_i}\right)$$

where *T* is the total number of cells, *t* is the number of neighbors, and *d*_*i*_ is the mean distance between center and target cells in the local neighborhood of cell *i*. For neighborhoods without valid center-target pairs, the SAI score was set to zero. SAI scores were stored in the cell metadata (adata.obs) for downstream visualization and statistical analysis. In detail, SAI scores were computed with the parameter n_neighbors set to 30 and stored in the cell metadata (adata.obs) for downstream visualization and statistical analysis. The resulting enrichment matrix was visualized using two complementary approaches: (i) dot plots, where both color intensity and dot size represent enrichment strength, and (ii) in situ scatter plots, where enrichment strength is reflected by color intensity.

### Trajectory inference

To reconstruct cellular trajectories and infer pseudotime progression, we applied the Palantir algorithm (V1.4.1) on the single-cell RNA-seq dataset [25]. After data normalization and dimensionality reduction via PCA, the top principal components were used to construct a diffusion map, capturing the intrinsic structure of the data. A start cell was defined based on the expression of known progenitor or early-state marker genes. Palantir was used to compute pseudotime values and differentiation potentials across the dataset. The resulting trajectory captured the continuum of cellular states and branch probabilities toward multiple terminal fates. Gene expression trends along pseudotime were smoothed using generalized additive models (GAMs), and dynamic genes associated with trajectory progression were visualized via heatmaps and trend plots.

### Integration of scRNA-seq with spatial transcriptomics

Tangram (V1.0.4) was employed to integrate single-cell RNA-seq data with spatial transcriptomics. Initial preprocessing was carried out using the pp_adatas function, followed by deconvolution via the map_cells_to_space function to project single-cell profiles onto spatial coordinates. The resulting mappings were used to infer cell type localization and spatial organization within the tissue.

### Kaplan–Meier survival analysis

Kaplan-Meier survival analysis was performed using the linfelines (v0.27.8) to investigate the association between ZFP36, THBS1 expression levels, and immune cell spatial aggregation (SAI) intensity and patient prognosis. Patients were stratified into high-expression and low-expression groups based on the median expression level or SAI score. Survival probabilities were estimated using the KaplanMeierFitter, and statistical differences between groups were evaluated using the log-rank test. Transcriptomic and clinical data from GC patients were retrieved from TCGA and ACRG cohorts [11, 26].

### Gene set scoring and spatial association analysis

To evaluate the immunoregulatory potential of THBS1^+^ CAFs, we obtained two public genesets, including GSE7460_TREG_VS_TCONV_ACT_UP and GSE25087_TREG_VS_TCONV_ADULT_UP from the MSigDB. Expression of these gene sets was calculated to enrichment scores per spatial spot using AUCell algorithm. THBS1⁺ CAFs were defined based on a pre-specified THBS1 expression threshold. The spatial co-localization was quantified using the Spatial Aggregation Index (SAI) and visualized in spatial coordinates to assess potential spatial coupling between THBS1 expression and Treg-related immune regulation.

### Cell-cell communication analysis

Cell-cell communication analysis was performed using the CellChat R package (V2.1.0), which utilizes a curated ligand-receptor interaction database to infer intercellular signaling. Communication probabilities between cell types were estimated using the “computeCommunProb” function, and low-confidence interactions were removed with “filterCommunication”. Pathway-level communication activity was quantified using “computeCommunProbPathway”, and overall interaction networks were summarized with the “aggregateNet” function.

### GO enrichment analysis

DEGs analysis was performed using the “scanpy.tools.rank_gene_groups” function in the Scanpy (V1.9.6), applying the Wilcoxon rank-sum test with Bonferroni correction. Genes with an adjusted *P* < 0.05 and log₂ fold change (log₂FC) > 0.2 were considered significantly differentially expressed. In selected comparisons, “scanpy.tools.rank_gene_groups” was used instead with a slightly relaxed threshold of log₂FC > 0.1 and *P* < 0.05. GO enrichment analysis of the resulting gene sets was conducted using the metascape [27].

### Laser-assisted crosslinking and immunoprecipitation sequencing (LACE-seq)

To identify RNA targets of the RNA-binding protein ZFP36, LACE-seq was performed. Briefly, cells were subjected to UV crosslinking to preserve RNA-protein interactions, followed by immunoprecipitation using a ZFP36-specific antibody. Co-precipitated RNA fragments were purified, converted into cDNA libraries, and subjected to high-throughput sequencing. Raw reads were aligned to the human reference genome (hg38) using bowtie (v2.5.4). Binding peaks were called using Piranha (v1.2.1) to detect high-confidence crosslink sites. Annotated peaks were mapped to transcript features using GENCODE V42, and candidate binding sites on the FN1 transcript were extracted for downstream analysis. Peak visualization was conducted using the matplotlib (v3.5.2).

### Motif scanning

To define potential post-transcriptional targets of ZFP36 in CAF, we integrated binding motif analysis with gene expression correlation. ARE motifs of the canonical “AUUUA” sequence (scanned as “AUUUA” in RNA; “ATTTA” when using DNA references) were scanned within the 3′ UTRs of all protein-coding genes defined by GENCODE v42 on hg38. Genes containing five or more AUUUA/ATTTA motifs were retained as candidates for ZFP36 binding. In parallel, Pearson correlation coefficients were computed between ZFP36 expression and all other genes across CAF subsets from single-cell transcriptomic data. Genes showing a negative correlation (Pearson’s r < -0.2) were selected. The intersection was subjected to multiple-testing control (Benjamini-Hochberg) and designated the high-confidence target set (FDR < 0.05). Visualization of target gene overlap was performed using correlation-motif scatter plots.

### CIBERSORT analysis

Immune cell composition was estimated using the CIBERSORT-ABS (absolute mode; permutations = 1,000) algorithm applied to gene expression matrices from TCGA STAD dataset. A predefined immune signature matrix was used to infer the relative proportions of various immune cell subsets. To investigate the relationship between the immune microenvironment and fibroblast-related gene expression, Pearson correlation analysis was conducted between the estimated immune cell fractions and selected CAF-associated genes.

### TF network inference

Regulatory network inference at the single-cell level was conducted using the pySCENIC pipeline (V0.12.1). TF-target gene associations were established based on co-expression analysis and motif enrichment, using the GRNBoost2 algorithm and regulatory motif information from the Cistrome database. Genomic regions spanning 500 bp upstream to 100 bp downstream of each transcription start site (TSS) were used to define candidate binding sites, based on the hg38 genome assembly. Significant TF-gene interactions (adjusted *P* < 0.01) were retained to construct cell-type-specific regulons. The activity of each regulon in individual cells was quantified using the AUCell algorithm, generating a cell-by-regulon activity matrix. For visualization, regulon activity scores were projected onto UMAP using matplotlib (V3.5.2), activity scores are reflected by color intensity.

### Statistical analysis

All statistical analyses were conducted in “scipy.stats”. For two-group comparisons, we used Student’s *t* test when normality and homoscedasticity held and Welch’s *t* test when variances were unequal. When distributional assumptions were not met, we used the Mann-Whitney U test (Wilcoxon rank-sum equivalent). Two-sided tests were used unless otherwise indicated. Exact *P* values are reported in the figures and text, and a threshold of *P* < 0.05 was considered statistically significant.

## Results

### GC pathological types exhibit distinct spatial structures, cellular compositions and immune landscapes

**Fig. 1:**
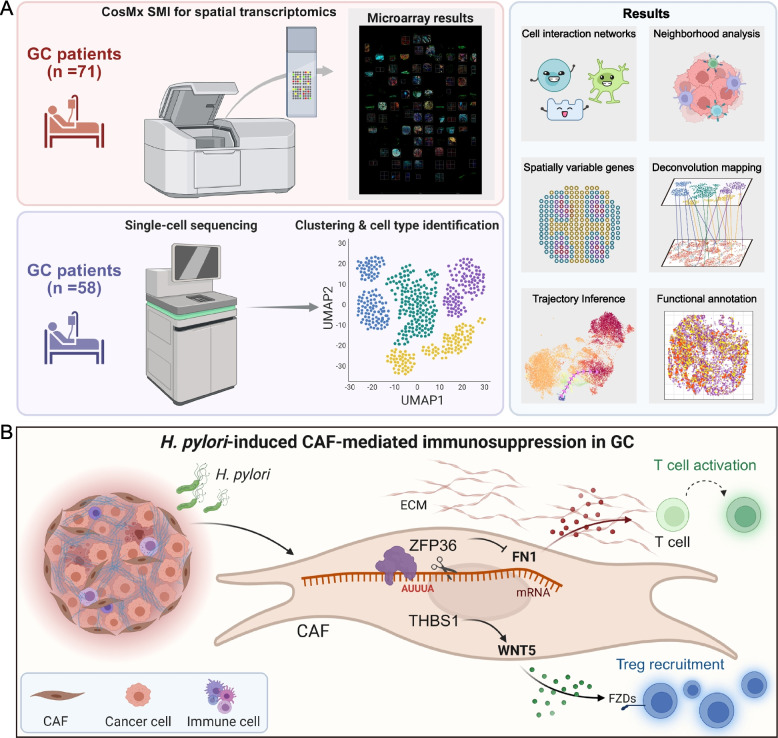
Schematic overview of CAF-mediated immunoregulation in *H. pylori*-associated GC. **A** Schematic representation of the integrated analytic framework used in this study. scRNA-seq and spatial transcriptomics were jointly applied to dissect CAF heterogeneity, spatial organization, cell-cell communication, trajectory dynamics, and functional states within the TME. Key analyses include neighborhood enrichment, interaction inference, pseudotime reconstruction, deconvolution-based spatial mapping, spatially variable gene detection, and functional annotation. **B** Graphical summary. Distinct CAF subtypes modulate immune responses via separate molecular axes: THBS1^+^ CAFs promote Treg recruitment and stabilization through the THBS1-WNT5 signaling axis, while ZFP36^+^ CAFs suppress cytotoxic lymphocyte engagement by downregulating FN1, collectively contributing to the formation of an “immune-cold” TME

To construct a spatially resolved atlas of the GC TME, we integrated scRNA-seq and spatial transcriptomics in human GC tissues. As demonstrated by the framework, we comprehensively analyzed cellular heterogeneity, spatial localization, intercellular communication, and lineage dynamics within the TME. Key computational steps included neighborhood enrichment, ligand-receptor inference, pseudotime analysis, spatial deconvolution, and functional annotation (Fig. 1A). In this study, *H. pylori* infection reshaped the tumor immune microenvironment in GC by transcriptionally reprogramming CAFs. The THBS1⁺ CAF subset aggregated near Tregs and might recruit Tregs through WNT5-FZDs signaling. Concomitantly, the RNA-binding protein ZFP36 bound to an AU (AUUUA)-rich element in the FN1 3′UTR, promoting FN1 mRNA degradation and possibly inhibiting CTL activation. Thus, the THBS1-WNT5 axis, which maintained Tregs, and the ZFP36-FN1 axis, which restricted CTL activation, might have acted together to drive localized immune suppression in *H. pylori*-positive GC, potentially revealing a viable stromal pathway for reversing immune evasion (Fig. 1B).

**Fig. 2:**
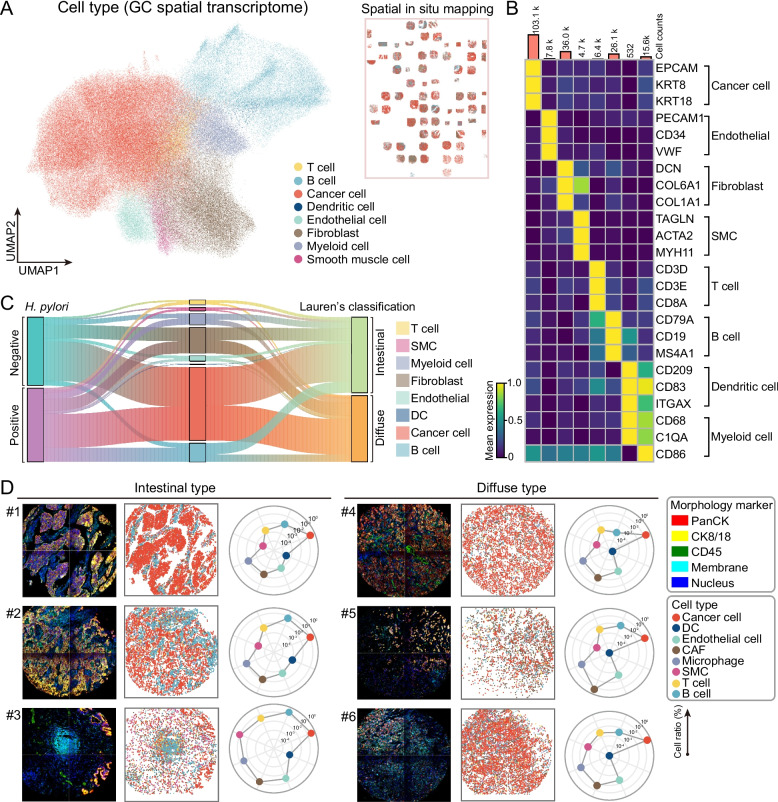
Spatial transcriptomic analysis reveals cellular composition and subtype-specific patterns in GC. **A** UMAP visualization of spatial transcriptomic spots colored by annotated cell types, including T cells, B cells, cancer cells, dendritic cells, endothelial cells, fibroblasts, myeloid cells, and smooth muscle cells. **B** Expression heatmap of representative marker genes used to define major cell types in spatial transcriptomic data. **C** Sankey plot illustrating the proportions of major cell types across GC subtypes defined by Lauren classification (intestinal vs. diffuse) and *H. pylori* infection status (positive vs. negative). **D** Representative spatial maps and quantification of cell type compositions in intestinal-type and diffuse-type GC samples. Radar charts summarize the relative abundance of each cell type across individual tumor samples

Guided by canonical markers, cell-type annotation of spatial transcriptomic data identified major immune, stromal, and malignant populations, including T cell, B cell, dendritic cell, myeloid cell, endothelial cell, fibroblast, smooth muscle cell, and malignant epithelial cell (Fig. 2A-B). We observed clear subtype-dependent differences in cell composition and spatial arrangement across Lauren-classified GC. In intestinal-type samples, cancer cells formed compact epithelial clusters, while CAFs and immune cells were enriched at the tumor margins, establishing well-demarcated tumor-stroma boundaries. In contrast, diffuse-type GC showed intermixed distributions of cancer cells, CAFs, and immune infiltrates, resulting in disorganized tumor architecture. Obviously, compared to diffuse-type GC sample, intestinal-type samples contained a higher proportion of immune cells compared to diffuse-type GC (Fig. 2C-D).

To further investigate whether stromal-immune coordination differed between subtypes, we performed cell-cell communication analysis. Interestingly, the intestinal-type GC samples exhibited more structured and directional signaling. In contrast, diffuse-type tumors showed enhanced yet dispersed communication, particularly involving fibroblasts, T cells, and myeloid cells (Fig. S1A). Moreover, diffuse-type GC displayed attenuated DC-derived signaling together with reduced T-cell communication compared to intestinal-type GC. This heatmap suggests stronger antigen-presenting activity in intestinal samples, but relatively restricted immune communication in diffuse tumors (Fig. S1B). At the ligand-receptor level, T-cell interactions were weaker in diffuse-type than in intestinal-type tumors (Fig. S1C-D). These findings indicate histology-specific immunoregulatory mechanisms. Taken together, our data demonstrate that GC subtypes differ markedly in both spatial organization and cell-cell communication.

### CAFs comprise four functional subtypes with distinct spatial distribution preferences

**Fig. 3:**
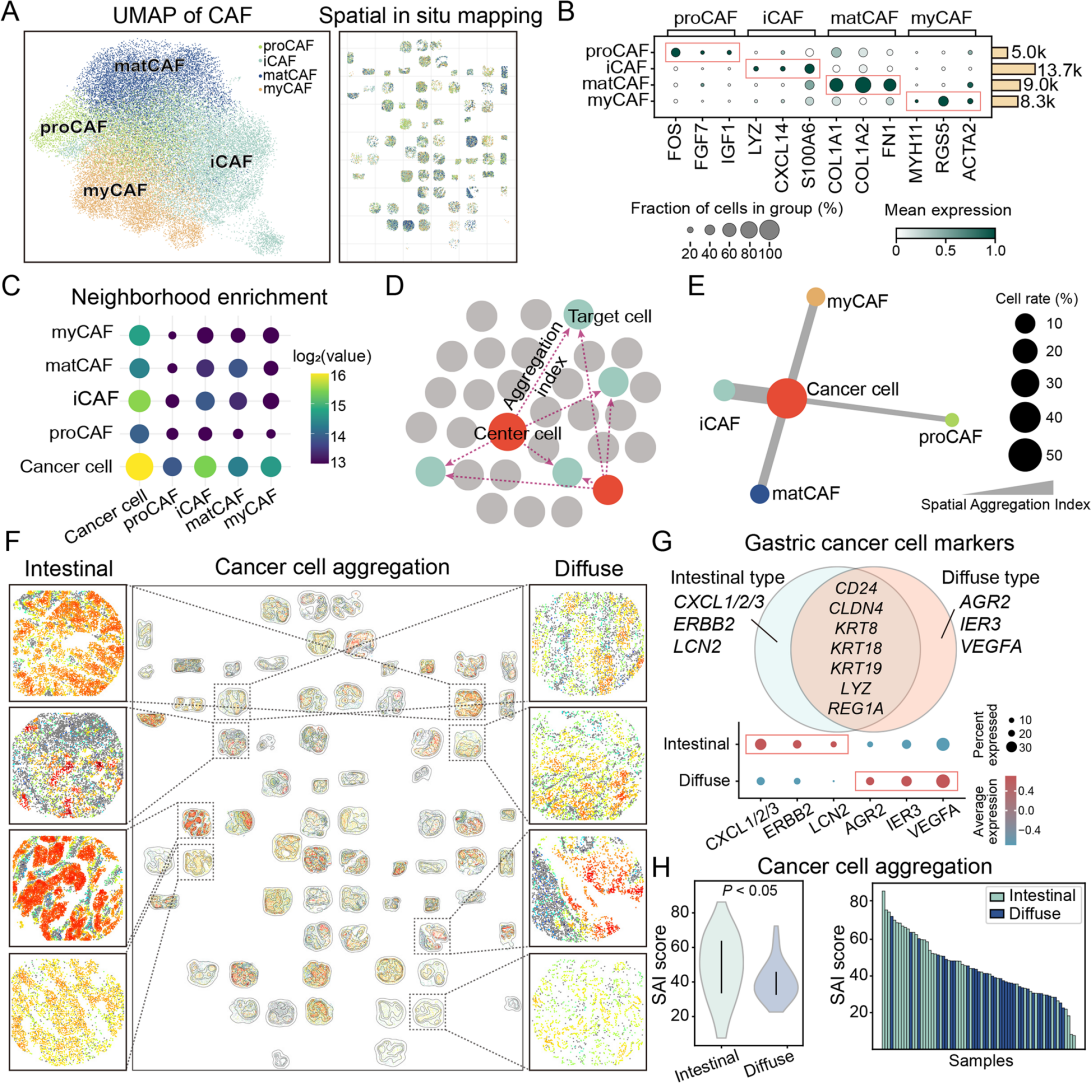
Spatial characterization of CAF subtypes and their spatial associations with cancer cells in GC. **A** UMAP visualization of spatial transcriptomic spots highlighting four CAF subtypes: proCAF, myCAF, iCAF, and matCAF. Corresponding spatial maps show the localization of these CAF subtypes across tissue sections. **B** Dot plot showing representative marker gene expression profiles for each CAF subtype. **C** Neighborhood enrichment analysis illustrating spatial proximity between each CAF subtype and cancer cells. **D** Schematic diagram of the aggregation index calculation used to quantify spatial proximity between cell types. **E** SAI score quantifying the degree of spatial clustering between cancer cells and each CAF subtype. **F** Spatial overview of cancer cell organization across multiple GC samples. Representative panels illustrate spatial differences in cancer cell distribution patterns across GC subtypes. **G** Marker gene expression distinguishing intestinal-type and diffuse-type GC cells. **H** Aggregation index comparing cancer cell clustering patterns between intestinal and diffuse subtypes

To investigate the spatial heterogeneity of CAFs in GC, we applied the classification scheme previously established in our pan-cancer single-cell dataset [18]. Using this framework, we spatially mapped four CAF subtypes: proCAFs, iCAFs, matCAFs, and myCAFs (Fig. 3A-B). This classification incorporated both gene expression profiles and spatial localization patterns. Each subtype displayed a distinct distribution across tissue sections, reflecting functional compartmentalization within the tumor microenvironment. Neighborhood enrichment analysis further revealed subtype-specific spatial associations with cancer cells. Among them, iCAFs localized in closest proximity to cancer cells (Fig. 3C). Consistently, quantitative modeling using the spatial aggregation index (SAI) confirmed this trend. iCAFs exhibited the strongest spatial proximity to cancer cells, followed by myCAFs and matCAFs, whereas proCAFs showed minimal spatial association (Fig. 3D-E).

Besides, using the SAI, we next assessed the clustering of cancer cells according to the Lauren classification. Spatial in situ mapping revealed subtype-specific aggregation patterns: intestinal-type tumors formed compact epithelial clusters, whereas diffuse-type tumors displayed dispersed distributions. These patterns corresponded to distinct stromal architectures and CAF-tumor spatial associations (Fig. 3F). Marker gene analysis further confirmed the molecular distinction between intestinal-type and diffuse-type GC (Fig. 3G). Consistently, SAI scores indicated significantly higher tumor cell clustering in intestinal-type tumors compared with diffuse-type tumors (*P* < 0.05; Fig. 3H). Together, these analyses highlight that intestinal- and diffuse-type GC differ markedly in both tumor cell organization and stromal context, supporting subtype-specific CAF-tumor interactions. These findings prompted further investigation into the developmental origins and regulatory mechanisms underlying CAF heterogeneity.

### Single-cell transcriptomics reveals developmental trajectories and transcriptional features of CAF subtypes

**Fig. 4:**
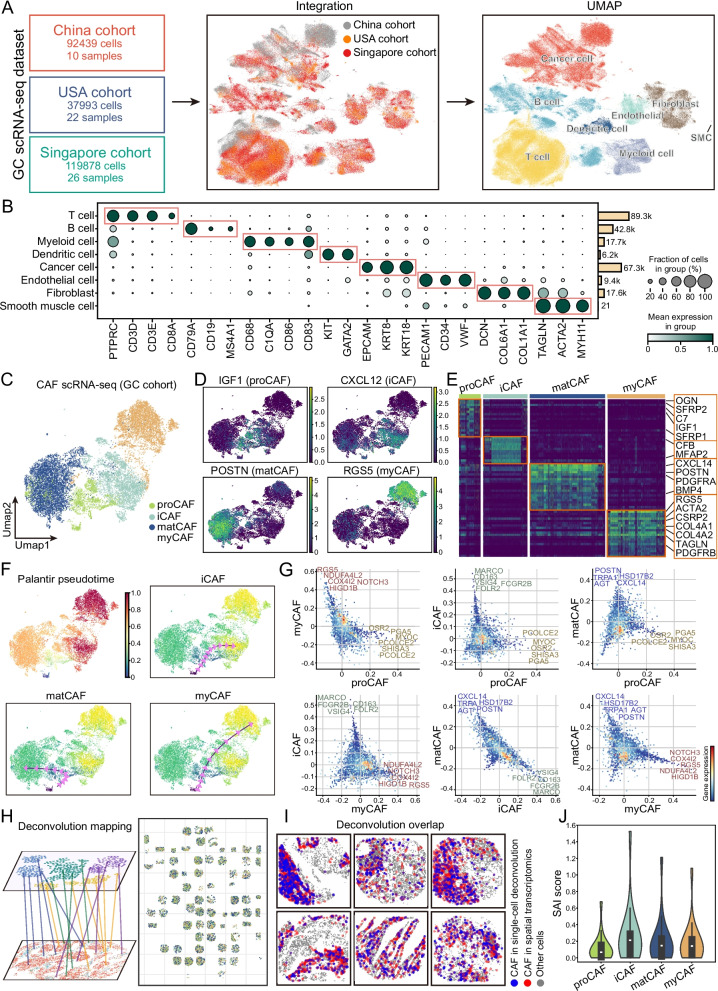
Single-cell transcriptomic analysis of CAF subtypes and their spatial dynamics in GC. **A** UMAP plots of GC samples from three cohorts (China, USA, and Singapore), integrated across datasets to visualize cellular heterogeneity. **B** Dot plot showing canonical marker gene expression for major cell types identified in the integrated scRNA-seq data. **C** UMAP visualization of four CAF subtypes identified from integrated scRNA-seq data: proCAF, myCAF, iCAF, and matCAF. **D** UMAP feature plots showing the expression of representative marker genes for each CAF subtype. **E** Heatmap displaying canonical marker gene expression patterns for each CAF subtype identified by scRNA-seq. **F** Pseudotime trajectory analysis of CAFs, indicating potential lineage progression across subtypes. **G** Pairwise comparisons of gene expression across CAF subtypes, with each dot representing a gene. **H** Deconvolution-based projection of single-cell-defined cell types onto spatial transcriptomic data. Left: schematic illustration of transcriptomic mapping from scRNA-seq to spatial coordinates; Right: spatial distribution of inferred cell types across tissue sections. **I** Overlap maps showing the spatial concordance between CAFs inferred from single-cell deconvolution (blue) and those identified in spatial transcriptomic data (red). Grey dots represent other cell types. **J** SAI score quantifying spatial clustering between each CAF subtype and cancer cells

To explore spatially relevant gene expression in greater depth, we integrated three single-cell RNA-seq datasets of GC, comprising 58 samples from China, the USA, and Singapore. In total, the dataset contained more than 250,000 cells. Using canonical marker genes, we performed cell-type annotation. Combined with marker gene expression, UMAP analysis confirmed the major cell populations, including cancer cells, T cells, B cells, dendritic cells, myeloid cells, endothelial cells, fibroblasts, and smooth muscle cells (Fig. 4A-B, Fig. S2). Obviously, the majority of fibroblasts could be subdivided into four CAF subtypes, proCAFs, iCAFs, matCAFs, and myCAFs, while a minor fraction represented normal fibroblasts. Each CAF subtype consistently expressed its canonical markers across the three cohorts (Fig. 4C-E). Pseudotime trajectory analysis revealed that proCAFs occupy an early developmental state. From this state, three distinct branches emerged, giving rise to iCAFs, matCAFs, and myCAFs. This pattern indicates multiple differentiation routes and functional specialization (Fig. 4F). Pairwise comparisons of gene expression programs further emphasized the transcriptomic divergence among CAF subtypes, underscoring their molecular distinctiveness and functional heterogeneity (Fig. 4G).

To link these transcriptomic identities with spatial context, we projected all single-cell-defined cell types onto spatial transcriptomic data using deconvolution, enabling tissue-level localization of CAFs and other key populations. The inferred spatial distributions closely matched those derived from direct spatial clustering ( Fig. 4H-I), validating the consistency of subtype localization. In the single-cell deconvolution analysis of CAF subtype recruitment to cancer cells, the strongest spatial aggregation of iCAFs with cancer cells, contrasted with the weakest association of proCAFs, corroborates our previous observation that iCAFs are positioned in closest proximity to cancer cells (Fig. 4J). These findings establish that CAF subtypes differ not only in spatial localization but also in developmental state and transcriptional regulation, highlighting coordinated transcriptional and spatial heterogeneity within the TME.

### H. pylori infection remodels CAF composition and induces THBS1 and ZFP36 expression in GC

**Fig. 5:**
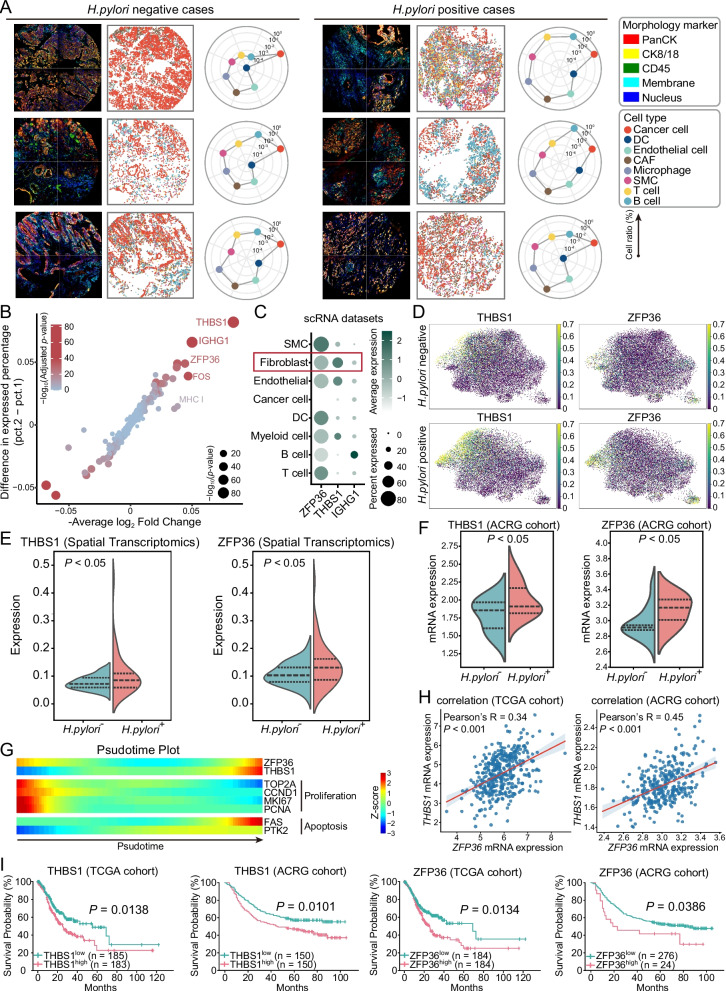
Expression dynamics and prognostic impact of THBS1 and ZFP36 in *H. pylori*-associated GC. **A** Representative spatial maps and quantification of cell type compositions in *H. pylori*-positive and *H. pylori*-negative GC samples. Radar charts summarize the relative abundance of each cell type across individual tumors based on spatial transcriptomic annotation. **B** Bubble plot showing differentially expressed genes between *H. pylori*-positive and *H. pylori*-negative tumors. Key upregulated genes in the *H. pylori*-negative group are highlighted. **C** Expression of selected genes (THBS1, ZFP36, IGHG1) across cell populations in scRNA-seq data. **D** UMAP visualization of THBS1 and ZFP36 expression in CAFs between *H. pylori*-positive and *H. pylori*-negative GC samples. **E** Violin plots comparing THBS1 and ZFP36 expression in CAFs between *H. pylori*-positive and *H. pylori*-negative tumors based on spatial transcriptomic data. **F** Violin plots showing mRNA expression levels of THBS1 and ZFP36 in *H. pylori*-positive and *H. pylori*-negative tumors from the ACRG cohort. **G** Pseudotime analysis showing dynamic expression changes of ZFP36, THBS1, and key proliferation- or apoptosis-related genes along the CAF differentiation trajectory. Expression is represented as Z-scored intensity across pseudotime. **H** Correlation analysis of THBS1 and ZFP36 mRNA expression levels in TCGA and ACRG GC cohorts. **I** Kaplan-Meier survival curves comparing overall survival in patients with high vs. low THBS1 or ZFP36 expressions in the TCGA and ACRG cohorts

A central question is whether *H. pylori* infection alters the stromal landscape of GC. Another, equally important, question is how such changes affect CAF composition and gene regulation. To address these issues, we analyzed spatial transcriptomic data from *H. pylori*-positive and *H. pylori*-negative GC samples. Spatial mapping revealed a significant increase in CAF abundance in infected tumors (Fig. 5A). Differential gene expression analysis identified THBS1 and ZFP36 as the most prominently upregulated genes in *H. pylori*-positive samples. Single-cell RNA-seq further confirmed that both genes were predominantly expressed in fibroblasts (Fig. 5B-C). Subtype-level analysis revealed that THBS1 expression was enriched in matCAFs, whereas ZFP36 was preferentially expressed in proCAFs (Fig. S3). Consistently, spatial transcriptomic visualization showed higher levels of THBS1 and ZFP36 in *H. pylori*-positive samples, supported by UMAP mapping and violin plot comparisons (Fig. 5D-E). Independent validation in the ACRG cohort reproduced these findings (Fig. 5F).

We next examined how these CAF-associated genes are positioned along fibroblast differentiation. Pseudotime trajectory analysis indicated that both THBS1 and ZFP36 expression increased toward terminal fibroblast states, where apoptosis-related genes such as FAS and PTK2 were also induced (Fig. 5G). Transcriptomic correlation analysis revealed a strong positive association between THBS1 and ZFP36 across both TCGA and ACRG datasets (Fig. 5H). Survival analysis demonstrated that high expression of either gene predicted significantly shorter overall survival (Fig. 5I). Taken together, these results indicate that *H. pylori* infection drives CAF expansion and induces transcriptional activation of THBS1 and ZFP36, especially in the proCAF subset.

### THBS1^+^ CAFs promote Treg recruitment and immune suppression via WNT5-FZDs ligand-receptor interactions

**Fig. 6:**
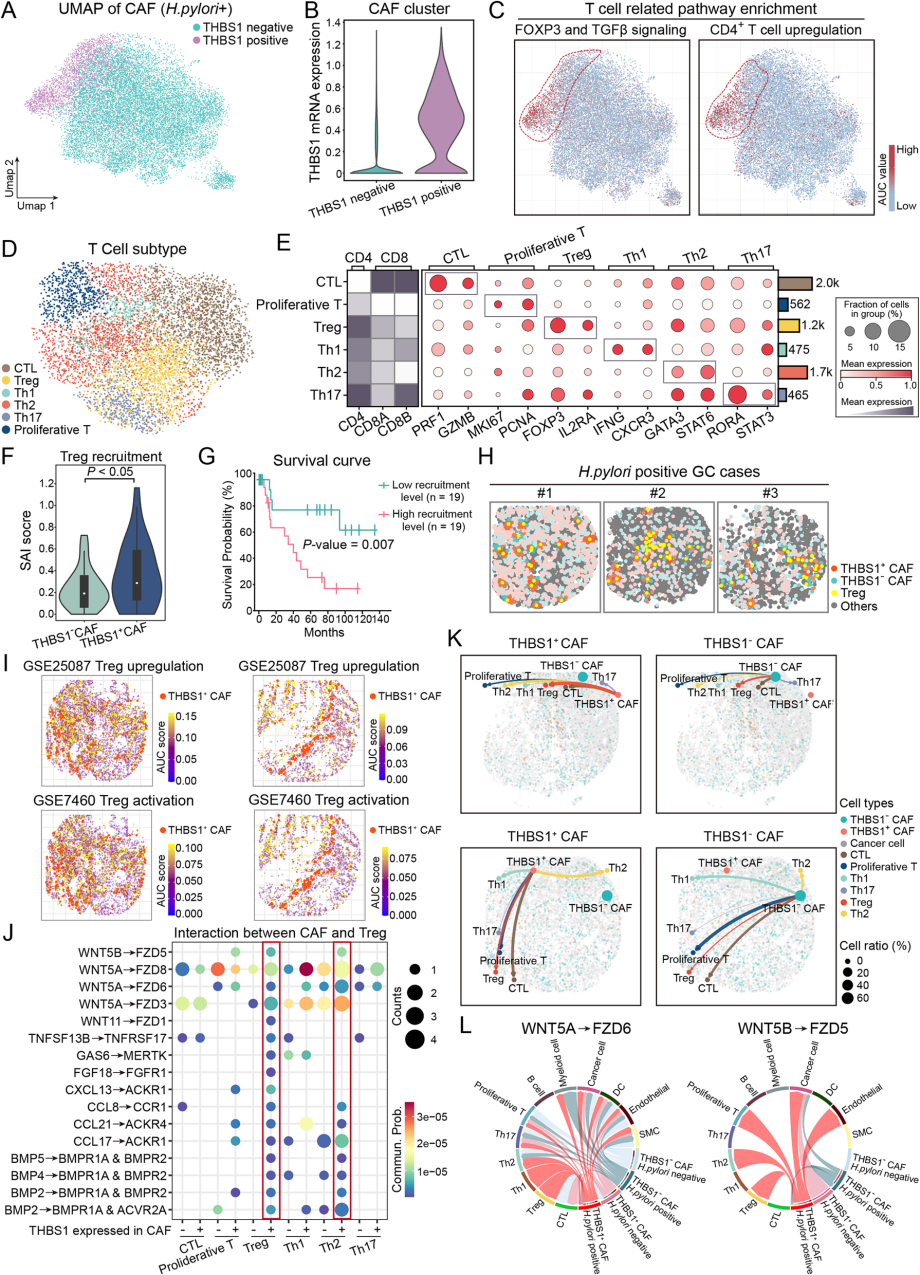
THBS1^+^ CAFs promote Treg recruitment and immune suppression in *H. pylori*-associated GC. **A** UMAP plot showing THBS1^+^ and THBS1^−^ CAFs identified from spatial transcriptomic data. **B** Violin plot validating the distinction between THBS1^+^ and THBS1^−^ CAF clusters based on THBS1 mRNA expression. **C** Enrichment analysis of T cell-related pathways (FOXP3/TGF-β signaling and CD4^+^ T cell upregulation) across the CAF population, showing localized enrichment patterns. **D** UMAP visualization of T cell subtypes identified in spatial transcriptomic data. **E** Dot plot showing canonical marker expression across major T cell subtypes, including CTL, Treg, Th1, Th2, Th17, and proliferative T cells. The heatmap on the left displays CD4, CD8A, and CD8B expression to assist in subset classification. **F** SAI score between THBS1^+^ and THBS1^−^ CAFs relative to surrounding Treg cells. **G** Kaplan-Meier survival curve comparing patients with low vs. high Treg recruitment in the presence of THBS1^+^ CAFs. **H** Spatial maps showing spatial distribution patterns of THBS1^+^ CAFs, THBS1^−^ CAFs, Tregs, and other cell types in *H. pylori*-positive GC samples. **I** Spatial enrichment analysis of Treg upregulation and activation pathways in GSE25087 and GSE7460 gene sets, highlighting spatial overlap with THBS1^+^ CAFs. **J** Dot plot showing predicted ligand-receptor interactions between THBS1^+^ CAFs and distinct T cell subtypes, with dot size indicating interaction frequency and color representing communication probability. **K** Spatially resolved cell-cell communication networks illustrating predicted interactions between THBS1^+^ or THBS1^−^ CAFs and T cell subsets in representative tumor sections. Arc thickness and color reflect inferred interaction strength; circle size indicates cell type abundance. **L** Chord diagrams depicting predicted WNT5A-FZD6 and WNT5B-FZD5 signaling interactions between THBS1^+^ or THBS1^−^ CAFs and other cell populations

To investigate the potential immunoregulatory role of THBS1⁺ CAFs in *H. pylori*-positive GC, we stratified CAFs based on THBS1 expression using spatial transcriptomic data. Distinct clusters of THBS1⁺ and THBS1⁻ CAFs were identified, with significant differences in THBS1 mRNA levels (Fig. 6A-B). Strikingly, THBS1⁺ CAFs were enriched for regulatory T cell (Treg)-associated signaling pathways, including FOXP3/TGF-β signaling and CD4⁺ T-cell activation pathways (Fig. 6C). We next characterized the T-cell compartment and identified six spatially resolved subpopulations: cytotoxic T lymphocytes (CTLs), Tregs, Th1, Th2, Th17, and proliferating T cells (Fig. 6D-E). Among these subsets, SAI analysis revealed a strong spatial association between Tregs and THBS1⁺ CAFs (Fig. 6F). Notably, tumors with high Treg enrichment in proximity to THBS1⁺ CAFs were associated with reduced overall survival (Fig. 6G). Representative images of the tumor microenvironment further illustrated this spatial relationship (Fig. 6H). Furthermore, we analyzed publicly available Treg-related gene signatures (GSE25087 and GSE7460). The spatial distribution of Treg gene activity partially overlapped with THBS1⁺ CAF localization ( Fig. 6I). Cell-cell communication analysis further predicted preferential ligand-receptor interactions between THBS1⁺ CAFs and Tregs. In particular, WNT5A-FZD6 and WNT5B-FZD5 signaling emerged as prominent interaction axes (Fig. 6J-L). Together, these results demonstrate that THBS1⁺ CAFs are spatially associated with Tregs and engage in WNT5-FZDs signaling, thereby contributing to the establishment of a locally immunosuppressive microenvironment in *H. pylori*-associated GC.

### ZFP36 suppresses FN1^+^ CAF-mediated cytotoxic T cell activation by post-transcriptional repression

**Fig. 7:**
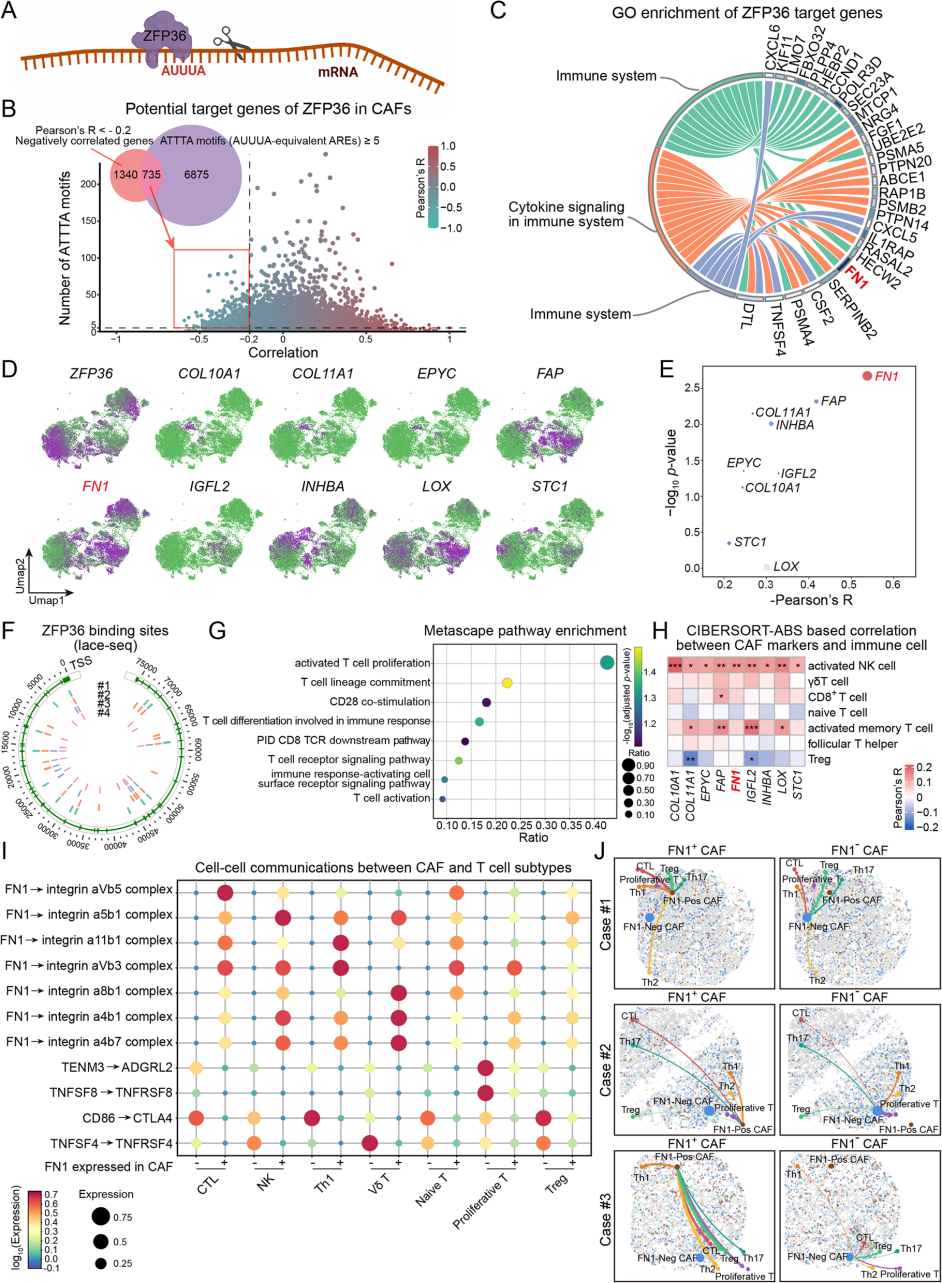
ZFP36 inhibits FN1^+^ CAF-mediated T cell activation via post-transcriptional repression in *H. pylori*-associated GC. **A** Schematic illustration of ZFP36-mediated post-transcriptional regulation. The diagram depicts ZFP36 binding to AU-rich elements (AUUUA motifs), within 3’UTRs to mediate mRNA degradation. **B** Scatter plot showing genes ranked by Pearson correlation with ZFP36 expression and the number of ATTTA motifs in their 3’UTRs, used as proxies for canonical AU-rich elements. Genes with Pearson’s R < -0.2 and ≥ 5 ATTTA motifs are highlighted as candidate ZFP36 targets. **C** Circular plot showing GO categories enriched among predicted ZFP36 target genes in CAFs. Immune system-related pathways and cytokine signaling terms are among the top-ranked categories. **D** UMAP feature plots showing expression patterns of ZFP36 and selected immune-related genes across CAF populations. **E** Scatter plot showing negatively correlated genes plotted by the strength of correlation with ZFP36 expression and statistical significance. **F** Circular plot depicting ZFP36 binding sites across the FN1 locus based on lace-seq data from four independent samples (#1-#4). TSS (transcription start site) is indicated, with binding peaks shown as colored bars corresponding to individual samples. **G** Bubble plot showing pathway enrichment results for ZFP36-associated target genes using Metascape analysis. Dot size represents the gene ratio, and color scale indicates adjusted *P*-values. **H** Heatmap showing correlations between selected CAF marker genes and immune cell types based on CIBERSORT-ABS analysis. Pearson’s correlation coefficients are represented by color gradients, with asterisks indicating statistical significance (^*^P < 0.05; ^**^P < 0.01; ^***^P < 0.001). **I** Bubble plot illustrating predicted ligand-receptor interactions between FN1^+^/FN1^−^ CAFs and T cell subtypes. Interaction strength is represented by dot size, and log_10_-transformed expression values are encoded by color. **J** Spatial cell-cell interaction networks comparing FN1^+^ and FN1^−^ CAFs across representative GC samples. Curved lines indicate predicted communication paths with various T cell subtypes, mapped within individual tissue sections

In addition to pathways associated with Treg recruitment, we next investigated whether other CAF subsets may engage distinct molecular programs involved in immune modulation. To this end, we examined upstream regulators of CAF-associated transcripts and focused on ZFP36, an RNA-binding protein known to mediate mRNA degradation via AU-rich elements (AREs), particularly AUUUA motifs, in 3’ untranslated regions (3'UTRs) (Fig. 7A). Correlation analysis across CAFs identified 735 candidate genes that were both negatively associated with ZFP36 expression (Pearson’s R < -0.2) and contained ≥ 5 ATTTA motifs, which are DNA/RNA equivalents of canonical AUUUA AREs (Fig. 7B). Gene ontology (GO) enrichment revealed that these targets were predominantly involved in immune-related pathways (Fig. 7C). Spatial feature plots showed distinct expression patterns of ZFP36 and selected immune-modulatory genes, including FN1, FAP, INHBA, COL10A1, and STC1 (Fig. 7D). Among these, FN1 demonstrated the strongest negative correlation with ZFP36 and ranked consistently among the top predicted targets (Fig. 7E). This regulation was further supported by direct evidence of ZFP36 binding to the FN1 3’UTR, as shown by LACE-seq data from four independent samples (Fig. 7F). Representative AU-rich motif-containing 3’UTR regions of FN1 with mapped ZFP36-binding sites are shown in Fig. S4, supporting direct post-transcriptional targeting.

To further explore the transcriptional context of ZFP36^+^ CAFs, we applied Single-Cell Regulatory Network Inference and Clustering (SCENIC)-based regulon analysis to infer transcription factor (TF) activity at the single-cell level. UMAP visualization revealed that ZFP36^+^ CAFs were enriched for transcriptional programs associated with ATF3, JUN, and FOS, factors linked to cellular stress and immune signaling (Fig. S5). These results suggest that ZFP36 may function in concert with specific transcriptional networks to modulate the immune-regulatory phenotype of CAFs. Consistently, pathway enrichment of ZFP36-associated targets highlighted roles in T cell activation and costimulatory signaling (Fig. 7G). To evaluate the immunological consequences of this regulation, we assessed correlations between FN1 expression and immune infiltration using CIBERSORT-ABS. FN1 expression in CAFs was positively associated with multiple T cell subsets, particularly activated CD8^+^ T cells and γδ T cells (Fig. 7H). To resolve the T cell compartment relevant to FN1^+^ CAF-mediated communication, we performed single-cell clustering of T cells and identified seven transcriptionally distinct subsets (Fig. S6A-B). Ligand-receptor modeling predicted that FN1^+^ CAFs engaged more extensively with T cells than FN1^−^ CAFs, via integrin-based and checkpoint-related interactions, such as α5β1-FN1, CD86-CTLA4, and TNFSF4-TNFRSF4 (Fig. 7I). Spatial cell-cell communication networks further demonstrated that, compared to FN1^−^ CAFs, FN1^+^ CAFs exhibited stronger spatial engagement with cytotoxic T cell subsets, including CTLs, across representative tumor sections (Fig. 7J). These results collectively demonstrate that ZFP36 post-transcriptionally represses FN1 and downregulates a CAF program associated with cytotoxic T cell activation, thereby contributing to immune modulation in *H. pylori*-associated GC.

## Discussion

In this study, we employed an integrated approach combining spatial transcriptomics and scRNA-seq to comprehensively characterize the CAF landscape in GC. Through the identification of distinct CAF subtypes, their spatial localization, and developmental trajectories, we uncovered key insights into their roles in GC pathogenesis. Notably, our data highlights the critical involvement of THBS1^+^ and ZFP36^+^ CAFs in modulating the immune microenvironment, particularly in the context of *H. pylori*-associated GC. These CAF subtypes were found to exert potent immunosuppressive effects by facilitating Treg recruitment and dampening effective anti-tumor immunity. Collectively, our findings position THBS1^+^ and ZFP36^+^ CAFs as pivotal mediators of immune evasion, offering novel perspectives on the mechanistic link between *H. pylori* infection, stromal remodeling, and GC progression.

It is well established that the composition and functional state of the TME are crucial drivers of GC progression and immune dynamics [28]. Among its stromal constituents, CAFs have been increasingly recognized as key modulators that actively regulate tumor growth, immune cell recruitment, and extracellular matrix remodeling [29, 30]. Histologically, we observed striking differences in the spatial organization of CAFs and immune cells between intestinal-type and diffuse-type GC. In the intestinal subtype, tumor cells formed compact clusters, with CAFs and immune cells predominantly localized at the tumor margins, thereby establishing well-defined structural boundaries. In contrast, diffuse-type GC displayed a more dispersed and intermixed pattern of tumor cells, CAFs, and immune infiltrates, consistent with its inherently invasive and non-cohesive morphology. Quantitative spatial metrics corroborated these observations, revealing significantly higher tumor clustering indices in the intestinal subtype. These findings are in line with the classic Lauren classification and provide a histological framework for understanding subtype-specific CAF distribution within the TME [7, 31]. Beyond structural differences, our cell-cell communication analysis revealed that intestinal-type tumors maintained more structured and directional signaling. In contrast, diffuse-type GC showed attenuated DC-derived signaling together with weaker T-cell interactions and dispersed fibroblast-immune crosstalk, indicating relatively restricted immune activity. In line with prior researches, our findings confirm the existence of histology-specific immunoregulatory mechanisms in GC [32, 33]. Taken together, these findings highlight histological subtype-specific immunoregulatory mechanisms, in which intestinal-type GC preserves coordinated stromal-immune communication, while diffuse-type tumors are characterized by disorganized signaling networks that may contribute to immune evasion.

Although iCAFs are often found in close proximity to cancer cells and engage in active stromal–immune crosstalk, ZFP36 and THBS1 were predominantly enriched in proCAFs. One potential explanation lies in the temporal sequence of *H. pylori*-driven stromal remodeling. *H. pylori* infection represents a well-characterized driver of early tumor-promoting inflammation and CAF activation in GC. As the progenitor subset, proCAFs are likely the first to be influenced by infection, acquiring immunomodulatory features such as ZFP36- and THBS1-mediated regulation that promote immune escape in the early stages of tumorigenesis. Our previous work demonstrated that iCAFs may emerge in large numbers following the induction of proCAFs, subsequently accumulating around cancer cells [18]. In this study, proCAFs act as the initial infection-responsive population carrying ZFP36 and THBS1 programs, while iCAFs represent a later expansion phase that spatially interacts with tumor cells and consolidates stromal-immune suppression. Collectively, these findings underscore the role of *H. pylori* as a multifaceted regulator of CAF activation, phenotypic plasticity, and functional specialization, providing a temporal-spatial perspective on how CAF subsets coordinate to establish immune evasion and drive GC progression.

Among these factors, *H. pylori* infection represents a well-characterized factor of tumor-promoting inflammation and stromal remodeling in GC. In our study, we observed a marked increase in the proportion of CAFs in *H. pylori*-positive tumors, consistent with previous findings including our own. Notably, we found that *H. pylori* infection leads to a marked increase in the proportion of CAFs within tumor samples, a finding that aligns closely with previous studies, including our own work. In our earlier work, we demonstrated that *H. pylori* activates key signaling cascades, specifically the NF-κB/PIEZO1/YAP1/CTGF axis, to drive CAF recruitment, facilitate extracellular matrix remodeling, and promote the development of an immunosuppressive TME [14]. These results are further supported by independent studies, which not only confirm CAF accumulation in *H. pylori*-positive tumors but also explore the molecular mechanisms underlying *H. pylori*-mediated CAF activation. Particularly, recent reports have shown that *H. pylori* induces the expression of immunomodulatory cytokines such as Serpin E1, which facilitate the transdifferentiation of normal fibroblasts (NFs) into CAFs, thereby enhancing tumor growth and angiogenesis [34, 35]. Collectively, these findings underscore the role of *H. pylori* as a multifaceted regulator of CAF activation, phenotypic plasticity, and functional specialization, offering deeper mechanistic insights into its contribution to immune modulation and GC progression.

At the molecular level, our analysis identified two key factors, THBS1 and ZFP36, that were significantly upregulated in CAFs from *H. pylori*-positive tumors. Importantly, elevated expression of both genes was strongly associated with poor clinical outcomes, as shown by significantly shorter overall survival in patients harboring THBS1^+^/ZFP36^+^ CAFs. For THBS1, its expression was spatially linked to Treg-enriched regions, suggesting a role in promoting Treg recruitment. Given its capacity to activate TGF-β signaling and suppress T-cell immunity via the CD47-SIRPα axis [36, 37], THBS1^+^ CAFs likely contribute to the establishment of an immunosuppressive microenvironment. Strikingly, patients with THBS1^+^ CAFs and low Treg recruitment exhibited approximately twofold higher survival rates compared to those with high Treg recruitment, underscoring the functional relevance of this interaction. To explore the underlying mechanism, we examined predicted cell-cell communication patterns and identified WNT-FZD interactions as the dominant signaling axis between THBS1^+^ CAFs and Tregs. While not a canonical chemotactic pathway, WNT signaling is known to stabilize Tregs and inhibit dendritic cell maturation to promote immune tolerance [38, 39]. These findings support a model in which a THBS1-WNT axis facilitates Treg accumulation and immunosuppressive niche formation in *H. pylori*-associated GC. Alternatively, the AU-rich element-binding protein ZFP36 may modulate CAF-mediated immune responses through a distinct mechanism [40, 41]. In our analysis, ZFP36 expression was inversely correlated with FN1, a key ECM component previously associated with immune exclusion via matrix stiffening and physical barriers to T-cell infiltration [42, 43]. Interestingly, our spatial interaction analysis revealed a different pattern: FN1^+^ CAFs were more frequently associated with CTLs and NK cells, whereas FN1^−^ CAFs were enriched for Treg interactions. These findings suggest that, in the context of *H. pylori*-associated GC, FN1 may contribute to immune activation, potentially by promoting interactions with cytotoxic immune cells [44, 45]. Given this, it is plausible that ZFP36 suppresses immunostimulatory CAF phenotypes by downregulating FN1, thereby complementing THBS1 in maintaining an immunosuppressive microenvironment through a separate regulatory axis.

Collectively, our integrated spatial and molecular profiling identifies THBS1^+^ and ZFP36^+^ CAFs as central orchestrators of immunosuppression and poor prognosis in *H. pylori*-associated GC. Specifically, the THBS1-WNT5 axis appears to promote Treg recruitment and stabilization, whereas the ZFP36-FN1 axis modulates immune exclusion and cytotoxic lymphocyte interactions, together contributing to the maintenance of an “immune-cold” TME. Nevertheless, these conclusions are largely based on transcriptomic correlations and require direct functional validation. Future studies involving CAF-specific gene perturbation and in vivo manipulation of THBS1 and ZFP36 will be critical to establishing causal relationships with immune modulation. These insights may provide a rationale for targeting stromal immune circuits in future immunotherapeutic strategies for *H. pylori*-associated GC.

## Conclusion

In conclusion, our study delineates the spatial and functional heterogeneity of CAF subtypes in GC and identifies *H. pylori* infection as a key external regulator of CAF immunomodulatory states. We propose two distinct CAF-mediated immune regulatory circuits, THBS1-WNT5-Treg and ZFP36-FN1, which collectively contribute to the establishment of an immunosuppressive TME in infection-associated GC. These findings provide novel mechanistic insights into stroma-immune-tumor interactions and offer a rationale for targeting CAFs as part of future immunotherapeutic strategies.

## Supplementary Material

Supplementary material 1.

## Data Availability

The datasets supporting this study are available from the following sources. The USA cohort scRNA-seq data are deposited in the Stanford DNA Discovery repository ([https://dna-discovery.stanford.edu/research/datasets/](https://dna-discovery.stanford.edu/research/datasets)). The Singapore cohort scRNA-seq data are available in NCBI GEO under accession GSE183904. Transcriptomic and clinical data used for survival analyses are obtainable from TCGA and ACRG (GEO GSE62254). The two public gene sets used for spatial enrichment analysis were obtained from MSigDB: GSE7460_TREG_VS_TCONV_ACT_UP ([https://www.gsea-msigdb.org/gsea/msigdb/human/geneset/GSE7460_TREG_VS_TCONV_ACT_UP.html](https://www.gsea-msigdb.org/gsea/msigdb/human/geneset/GSE7460_TREG_VS_TCONV_ACT_UP.html)) and GSE25087_TREG_VS_TCONV_ADULT_UP ([https://www.gsea-msigdb.org/gsea/msigdb/human/geneset/GSE25087_TREG_VS_TCONV_ADULT_UP.html](https://www.gsea-msigdb.org/gsea/msigdb/human/geneset/GSE25087_TREG_VS_TCONV_ADULT_UP.html)). The GC spatial dataset is deposited in GEO under accession GSE308624. The GC cell-line LACE-seq dataset is deposited in GEO under accession GSE309051. The implementation and analysis scripts of the Spatial Aggregation Index (SAI) are available on GitHub (https://github.com/AI-Lab-Pathology/SAI).

## Abbreviations

ACRG: Asian Cancer Research Group
CAF: Cancer-associated fibroblast
CTL: Cytotoxic T lymphocyte
DEG: Differentially expressed gene
FFPE: Formalin-fixed paraffin-embedded
GC: Gastric cancer
GO: Gene Ontology
HIER: Heat-induced epitope retrieval
iCAF: Inflammatory CAF
LACE-seq: Laser-assisted crosslinking and immunoprecipitation sequencing
matCAF: Matrix CAF
myCAF: Myofibroblastic CAF
PCA: Principal component analysis
proCAF: Progenitor CAF
scRNA-seq: Single-cell RNA sequencing
SCENIC: Single-Cell Regulatory Network Inference and Clustering
TCGA: The Cancer Genome Atlas
TF: Transcription factor
TME: Tumor microenvironment
Treg: Regulatory T cell
TSS: Transcription start site
UMAP: Uniform Manifold Approximation and Projection
UTR: Untranslated region
