# Supplementary material for: Spatial and functional dissection of cancer-associated fibroblasts-mediated immune modulation in H. pylori-associated gastric cancer
Source: Mol Cancer. 2025 Nov 6;24:282. doi: 10.1186/s12943-025-02490-9 (PMC12590883; doi:10.1186/s12943-025-02490-9)
Supplement: Supplementary file 1 — Supplementary material 1. [file 12943_2025_2490_MOESM1_ESM.pdf]

Supplemental materials for

Spatial and functional dissection of cancer-associated fibroblasts-mediated immune modulation in *H. pylori*-associated gastric cancer

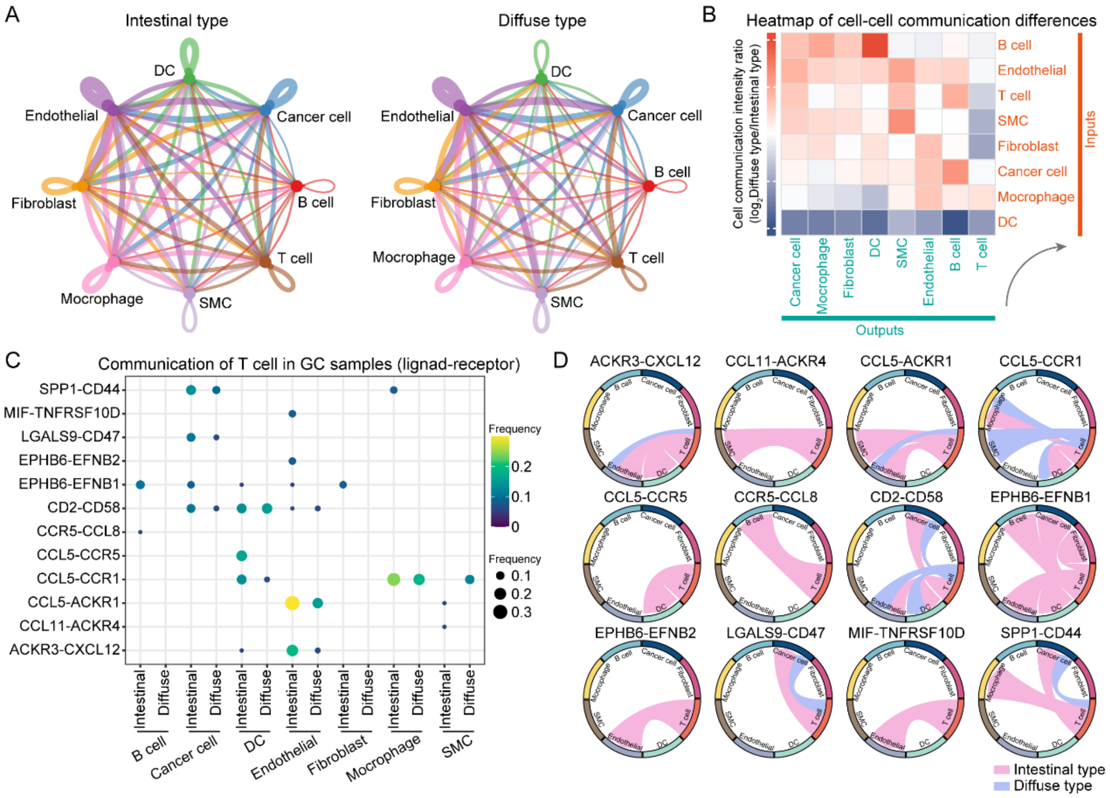

Figure S1. Subtype-specific cell-cell communication landscape in GC. (A) Circle plots showing the inferred intercellular communication networks among major cell types based on ligand-receptor interactions in intestinal and diffuse subtypes of (GC). Line width reflects the total number of significant interactions between sender and receiver cells. (B) Heatmap summarizing the relative communication intensity differences (log_2 fold change) between the two subtypes**,** across all sender-receiver cell pairs. Red and blue indicate stronger interactions in intestinal and diffuse tumors, respectively. (C) Bubble plot showing ligand-receptor communication events between

T cells and other cell types in each GC subtype. Each dot represents a ligand-receptor pair, with dot size and color indicating communication frequency across samples. (D) Chord diagrams visualizing representative ligand-receptor interactions involving T cells in the two subtypes. Each panel illustrates the direction and strength of communication between specific sender and receiver cell types for individual ligand-receptor pairs. Pink and blue arcs represent interactions enriched in intestinal-type and diffuse-type GC, respectively.

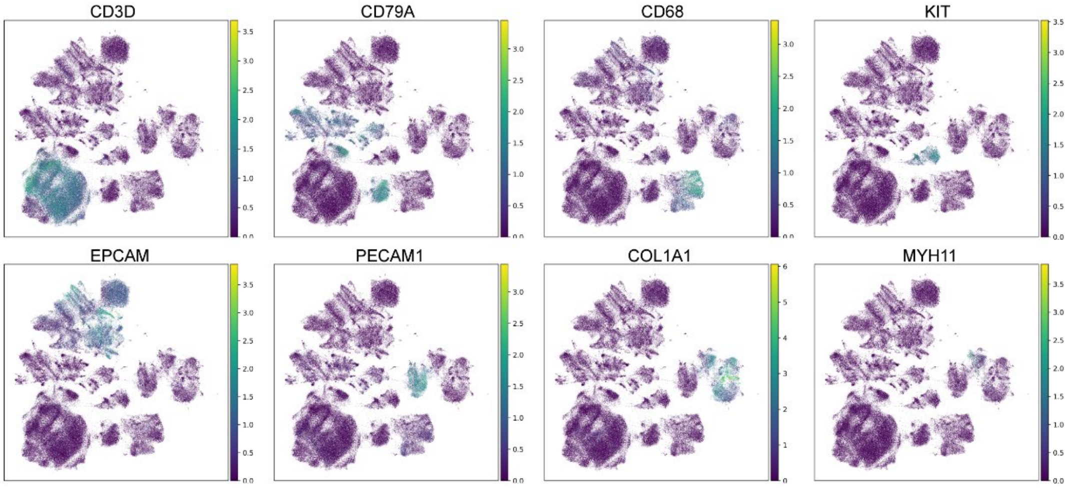

Figure S2. Expression of cell type marker genes in the scRNA-seq dataset. The feature plot shows the expression patterns of representative marker genes for major cell types in the integrated gastric cancer (GC) scRNA-seq dataset, including CD3D (T cells), CD79A (B cells), CD68 (myeloid cells), KIT (mast cells), EPCAM (cancer cells), PECAM1 (endothelial cells), COL1A1 (fibroblasts), and MYH11 (smooth muscle cells).

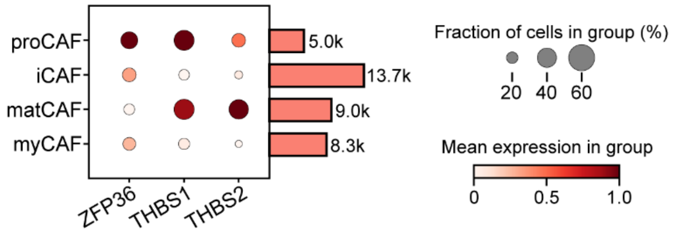

Figure S3. Expression patterns of ZFP36, THBS1, and THBS2 across cancer-associated fibroblast (CAF) subtypes. Dot plot showing the mean expression (color intensity) and percentage of expressing cells (dot size) for ZFP36, THBS1, and THBS2 across four CAF subtypes, including proCAF, iCAF, matCAF, and myCAF.

chr2:215361349-	TGGAAAGATGATTTACTCTCGGGAA	-chr2:215361564	chr2:215376291-	ACACTGAAAATAAATTTTATCAGTG	-chr2:215376506
chr2:215391476-	CTACTTGAGAATTTAGAATTCATTT	-chr2:215391691	chr2:215391476-	CTAGGTTAATATTTATGGAACAGAT	-chr2:215391691
chr2:215392627-	GCTATTTTATATTTAACAGAGCAGA	-chr2:215392842	chr2:215392627-	ACTTGTGGTTTAAATGAGAGTGTAC	-chr2:215392842
chr2:215404176-	AGTATCACTGATTTAAATTATGTAC	-chr2:215404391	chr2:215404176-	ATCACTGATTTAAATTATGTACTAA	-chr2:215404391
chr2:215404176-	ATAGAGAATGTAAATATAGTTAAGA	-chr2:215404391	chr2:215407612-	TCCTTAAGGATAAATCTTAGAGCAA	-chr2:215407827
chr2:215370176-	AGCCTGTGTCTAAATAGTACGTGTT	-chr2:215370391	chr2:215378990-	GAACAGTTAGATTTATTATGATATT	-chr2:215379205
chr2:215380604-	CTTGTTTCCTTAAATATCACCTAAG	-chr2:215380819	chr2:215414673-	ACAAATATAGTAAATGCTATAGGAA	-chr2:215414888
chr2:215423225-	CTCTACTCCCTAAATTGTTGTCAAA	-chr2:215423440	chr2:215423967-	GGGTTCAAAATAAATGGCTAGAATG	-chr2:215424182
chr2:215362080-	GGACATCGTAATTTAGTGTTTGAGT	-chr2:215362295	chr2:215362080-	GATTTCATGCATTTATAACTCTTAA	-chr2:215362295
chr2:215406971-	AACTTCTGATATTTAGCTGAAATGT	-chr2:215407186	chr2:215361328-	TGCAGCCCTCATTTATGAGAAAACC	-chr2:215361543
chr2:215427968-	ACCTAAGATAATTTACACATTATTG	-chr2:215428183	chr2:215427968-	TGTCAAGTTTTAAATTTTTCAGAAA	-chr2:215428183

Figure S4. LACE-seq identified representative AUUUA-containing regions in the FN1 3’UTR. The selected sequences include canonical ZFP36-binding motifs with surrounding context, and genomic coordinates are provided. These features suggest potential post-transcriptional regulation of FN1 by ZFP36.

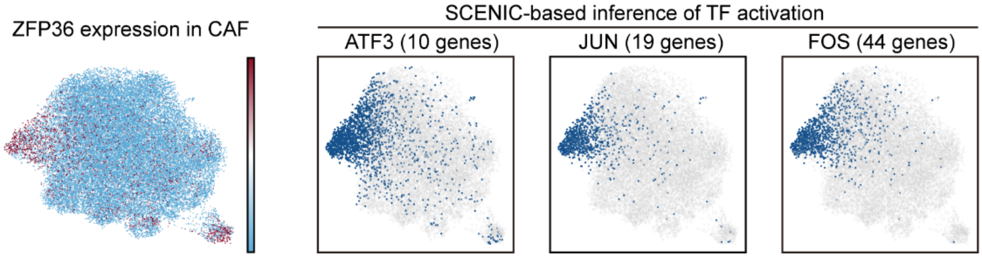

Figure S5. Transcriptional activity associated with ZFP36 expression in CAFs. Left: UMAP plot showing the expression pattern of ZFP36 across CAF populations. Right: SCENIC-based transcription factor activity inference indicating that high ZFP36 expression is associated with increased activity of ATF3, JUN, and FOS regulons, based on target gene module enrichment scores.

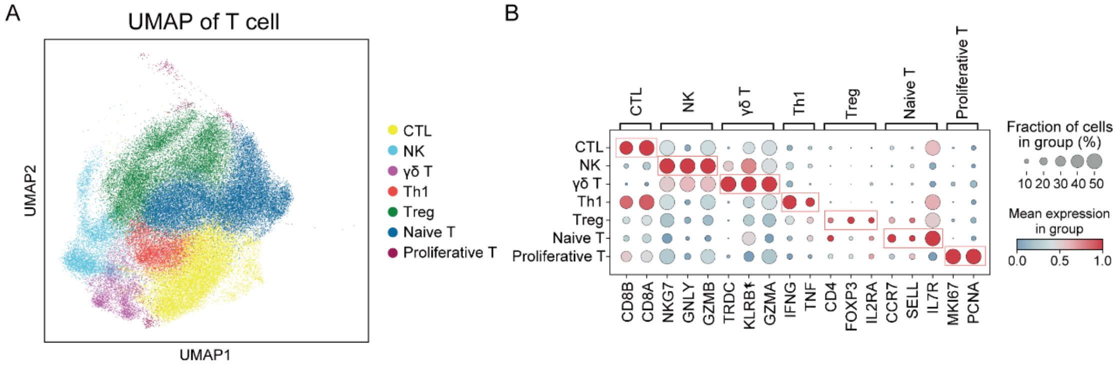

Figure S6. Single-cell characterization of T cell subsets in GC. (A) UMAP visualization of T cells from integrated scRNA-seq data, identifying seven transcriptionally distinct subsets, including CTL, NK, γδ T, Th1, Treg, naive T, and proliferative T cells. (B) Dot plot showing representative marker gene expression across the T cell subsets. Dot size indicates the proportion of cells expressing the gene in each cell type, and color reflects average expression level.
